# Smartphone-Based Interventions to Reduce Sedentary Behavior and Promote Physical Activity Using Integrated Dynamic Models: Systematic Review

**DOI:** 10.2196/26315

**Published:** 2021-09-13

**Authors:** Reza Daryabeygi-Khotbehsara, Sheikh Mohammed Shariful Islam, David Dunstan, Jenna McVicar, Mohamed Abdelrazek, Ralph Maddison

**Affiliations:** 1 Institute for Physical Activity and Nutrition Deakin University Geelong Australia; 2 Physical Activity Laboratory Baker Heart and Diabetes Institute Melbourne Australia; 3 Behaviour, Environment and Cognition Research Program, Mary MacKillop Institute for Health Research Australian Catholic University Melbourne Australia; 4 School of Information Technology Deakin University Geelong Australia

**Keywords:** smartphone, mobile phone, physical activity, sedentary behavior, computational models, control systems, systematic review

## Abstract

**Background:**

Traditional psychological theories are inadequate to fully leverage the potential of smartphones and improve the effectiveness of physical activity (PA) and sedentary behavior (SB) change interventions. Future interventions need to consider dynamic models taken from other disciplines, such as engineering (eg, control systems). The extent to which such dynamic models have been incorporated in the development of interventions for PA and SB remains unclear.

**Objective:**

This review aims to quantify the number of studies that have used dynamic models to develop smartphone-based interventions to promote PA and reduce SB, describe their features, and evaluate their effectiveness where possible.

**Methods:**

Databases including PubMed, PsycINFO, IEEE Xplore, Cochrane, and Scopus were searched from inception to May 15, 2019, using terms related to mobile health, dynamic models, SB, and PA. The included studies involved the following: PA or SB interventions involving human adults; either developed or evaluated integrated psychological theory with dynamic theories; used smartphones for the intervention delivery; the interventions were adaptive or just-in-time adaptive; included randomized controlled trials (RCTs), pilot RCTs, quasi-experimental, and pre-post study designs; and were published from 2000 onward. Outcomes included general characteristics, dynamic models, theory or construct integration, and measured SB and PA behaviors. Data were synthesized narratively. There was limited scope for meta-analysis because of the variability in the study results.

**Results:**

A total of 1087 publications were screened, with 11 publications describing 8 studies included in the review. All studies targeted PA; 4 also included SB. Social cognitive theory was the major psychological theory upon which the studies were based. Behavioral intervention technology, control systems, computational agent model, exploit-explore strategy, behavioral analytic algorithm, and dynamic decision network were the dynamic models used in the included studies. The effectiveness of quasi-experimental studies involved reduced SB (1 study; *P*=.08), increased light PA (1 study; *P*=.002), walking steps (2 studies; *P*=.06 and *P*<.001), walking time (1 study; *P*=.02), moderate-to-vigorous PA (2 studies; *P*=.08 and *P*=.81), and nonwalking exercise time (1 study; *P*=.31). RCT studies showed increased walking steps (1 study; *P*=.003) and walking time (1 study; *P*=.06). To measure activity, 5 studies used built-in smartphone sensors (ie, accelerometers), 3 of which used the phone’s GPS, and 3 studies used wearable activity trackers.

**Conclusions:**

To our knowledge, this is the first systematic review to report on smartphone-based studies to reduce SB and promote PA with a focus on integrated dynamic models. These findings highlight the scarcity of dynamic model–based smartphone studies to reduce SB or promote PA. The limited number of studies that incorporate these models shows promising findings. Future research is required to assess the effectiveness of dynamic models in promoting PA and reducing SB.

**Trial Registration:**

International Prospective Register of Systematic Reviews (PROSPERO) CRD42020139350; https://www.crd.york.ac.uk/PROSPERO/display_record.php?RecordID=139350.

## Introduction

In the past decade, there has been a widespread proliferation in the use of digital technologies to deliver behavior change interventions for health [1]. Given their ubiquity, smartphones, in particular, have been used to improve a wide range of health-related behaviors, including physical activity (PA) and sedentary behavior (SB) [2,3]. Smartphones offer a host of relevant functions, including computational capabilities, built-in sensors (eg, accelerometers and GPS), and internet connectivity, enabling users to run software apps and connect with third-party sensors. Collectively, these features offer the potential for delivering real-time, context-aware, and interactive health care interventions [4].

Theory-based lifestyle interventions have been shown to be more effective than nontheoretical approaches [5]. Thus, to better leverage the potential of mobile technologies for health behavior interventions (mobile health [mHealth]), appropriate behavior change theories and models are needed. Such theories and models need to guide the design and development of complex smartphone interventions that can adapt rapidly in response to various inputs [4]. To date, many smartphone-based interventions to promote PA and reduce SB have relied predominantly on psychological theory, including social cognitive theory (SCT) and self-efficacy theory [2,6]. In a seminal paper, Riley et al [4] argued that current behavioral theories do not meet the need for a more dynamic and interactive nature of digital behavior change interventions, such as just-in-time adaptive interventions. These just-in-time adaptive interventions are complex interventions that adapt throughout time to an individual’s time-varying context (where) and status (when) to meet an individual’s changing needs for support [7-9]. Riley et al [4] argued that existing psychological theories are relatively static and linear and lack sufficient within-subject dynamic regulatory processes. Furthermore, current psychological theories have been used to tailor interventions based on preintervention data rather than deliver adaptive interventions.

To transform current theories into dynamic frameworks and fully maximize the potential of smartphone technologies, Riley et al [4] highlighted the need to incorporate theories from other disciplines (eg, computer science and engineering) for the future development of adaptive and dynamic digital behavior change interventions. One such theory is the control systems theory—derived from the *control theory* or *cybernetics*—which is a general concept for the understanding of regulatory processes [10] and has various applications in engineering, mathematics, medicine, and economics, among others. Control systems engineering explores how to influence and regulate a dynamic system (eg, time-varying adaptive PA intervention) [11,12]. Applying these dynamic models to health behaviors offers the potential to better predict behavior and provide greater insight into real-time changes, which, in turn, enable the optimization and maintenance of behaviors [9].

Since the study by Riley et al [4] was published, it is unclear how many smartphone-based interventions targeting PA and SB have integrated nonpsycholgical theories to create more dynamic models for digital behavior change interventions, what adaptive factors have been considered, and whether these dynamic interventions improve behaviors. Therefore, this review aims to (1) quantify the number of studies that have used integrated dynamic models to develop smartphone-based interventions to promote PA and reduce SB, (2) describe their features, and (3) evaluate their effectiveness, where possible.

## Methods

### Design

The systematic review was performed according to the PRISMA (Preferred Reporting Items for Systematic Reviews and Meta-Analyses) statement [13] and was registered with PROSPERO (International Prospective Register of Systematic Reviews; CRD42020139350) [14].

### Study Criteria

This review included studies that developed or evaluated digital behavior change interventions targeting PA, SB, or both and integrated psychological theories with dynamic theories and computational models (eg, control systems engineering); were either adaptive or just-in-time adaptive interventions that included smartphones for delivery; involved human adult participants; included randomized controlled trials (RCTs), pilot RCTs, quasi-experimental, pre-post study designs; and were published from 2000 onward.

### Exclusion Criteria

Studies that used conventional theories of behavior change alone without integration with dynamic theories or computational models, case studies, protocols, conference abstracts, dissertations, and reviews were excluded.

### Definition

For this review, *dynamic theories* refer to dynamic models taken from other disciplines, including engineering (eg, control systems engineering) and computer science (eg, agent-based modeling). The defining features of dynamic approaches are that they are not static, nonlinear in nature, and capable of capturing complex and rapid changes in behaviors (ie, time-variant) and their influential factors (ie, multivariate). Furthermore, they are quantifiable, empirical, and testable models.

### Search Strategy

Databases (IEEE, PubMed, PsycINFO, Cochrane, and Scopus) were searched from January 2000 to May 15, 2019, without language restriction. Keywords (including Medical Subject Headings terms) and phrases comprised 3 components (mHealth, dynamic models, and activity), where “OR” and “AND” Boolean operators were used for within and between component searching (Multimedia Appendix 1). The wild-card term “*” was used where necessary to potentiate sensitivity. Snowball searching was performed using the included studies to identify additional relevant research. The search results were exported to a reference manager software (EndNote X9; Clarivate Analytics) for review and extraction.

### Screening Process and Data Extraction

Two researchers (JMV and RDK) independently screened and reviewed the titles and abstracts to identify eligible studies. The full text of the included papers was assessed based on the study criteria. The following information was collected: author and year, country, study design, duration of the study, recruitment and setting, the population of the study, sample size, inclusion criteria, participant characteristics, dynamic model, theory or constructs integrated, and outcomes measured (SB and PA behaviors).

### Quality Assessment

Two researchers (JMV and RDK) assessed the risk of bias. The Cochrane Handbook for Systematic Reviews of Interventions [15] was used to evaluate randomized studies for selection bias, detection bias, attrition bias, performance bias, and reporting bias as the main sources of bias. Other sources of bias were also considered. In addition, the Joanna Briggs Institute Critical Appraisal Checklist for Quasi-Experimental Studies [16] was used to assess nonrandomized studies. Where available, protocols and trial registry data were found for risk of bias assessment. Where multiple reports existed for the same study, data were extracted from all reports and expressed together. The authors were contacted for further information, as needed.

### Data Analysis

The data were synthesized narratively to address the aims of this review. Given the heterogeneity of the included studies in terms of methodology, outcome measures, and statistical approaches, a meta-analysis of effectiveness data was not conducted. Instead, a synthesis without a meta-analysis method—vote counting based on the direction of effects—was used to synthesize data [17]. The effect direction is a standardized binary metric based on the observed benefit (positive) or harm (negative). Vote counting is based on effect direction and compares the number of positive effects with the number of negative effects on an outcome. An effect direction plot is used for the visual representation of data and linking narrative synthesis to the overall conclusion [18,19]. In this review, the updated method of the effect direction plot is used as outlined elsewhere [20]. Changes within the intervention arm of controlled studies and changes from baseline in uncontrolled studies were considered for judgment. PA outcomes including light activity, walking (time and steps), moderate-to-vigorous PA (MVPA), nonwalking exercise, and total PA time from 6 studies were grouped as PA health domains. For studies with multiple PA outcomes, the effect direction was where 70% or more of the outcomes reported a similar direction (positive or negative). If less than 70% of outcomes showed a similar direction, they were reported as conflicting findings or no clear effect. A sign test was applied to test any evidence of an effect across studies. A 2-tailed *P* value was then calculated to show the probability of observing positive and negative findings for the PA health domain.

## Results

### Overview

**Figure 1:**
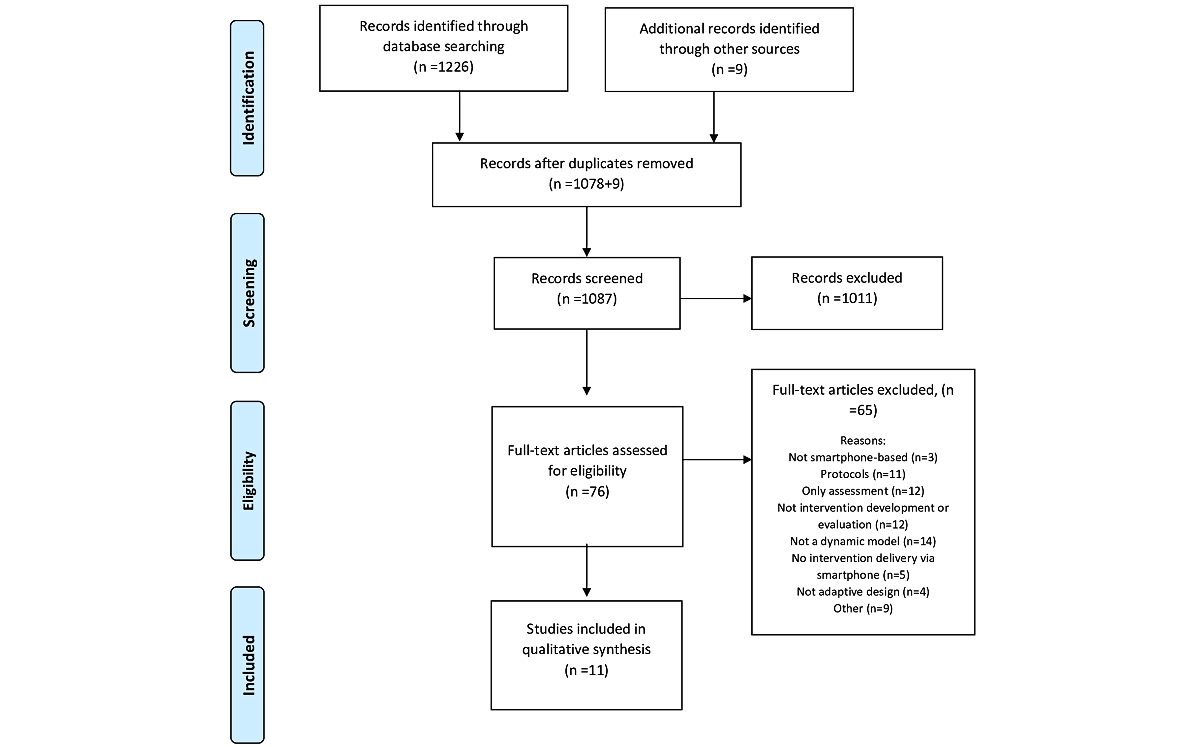
Flow of studies. PRISMA: Preferred Reporting Items for Systematic Reviews and Meta-Analyses [13].

A total of 1087 study reports were identified after removing duplicates. In addition, 9 studies were identified through a manual search. A total of 76 research articles underwent a full review, and 11 reports describing 8 studies were eligible and included in this systematic review. The characteristics of the included studies are summarized in Table 1. The inclusion process and reasons for exclusion are shown in the PRISMA flow diagram (Figure 1). The reasons for excluding 65 studies (in full-text review) are provided in Multimedia Appendix 2 [21-85].

**Table 1:** General characteristics of the included studies.

Author (year)	Country	Study design and duration	Recruitment setting	Sample, n	Inclusion criteria	Participants’ characteristics
Baretta et al (2019) [86]	Italy	Intervention development; 8 weeks	Indoor activity settings (eg, gyms)	60	Not described	People who did not meet PAa guidelines Age (35-60 years) Female (35/60, 58%)
Direito et al (2019) [87] (other related reference: Direito et al [88])	New Zealand	Pre-post single-arm intervention; 8 weeks	Community	69	17-69 years Owning an Android phone	Insufficiently active healthy adults (either those who did not meet PA recommendations or who intended to decrease sedentary behavior) Mean age 34.5 (SD 11.8) years Female (54/69, 78%) Mean BMI 25.6 (SD 4.95) kg/m2 Ethnicity: New Zealand European (38/69, 55%)
Conroy et al (2018) [12]	United States	Single-group microintervention; 16 weeks	Community (via advertisement)	10	Adults not meeting federally recommended levels of aerobic PA but had no contraindications to PA	Mean age 34.4 (SD 9.0) years Female (9/10, 90%) Employed full time (8/10, 80%) Parents (6/10, 60%) Single (5/10, 50%), married (4/10, 40%), or divorced (1/10, 10%) Education (6/10, 60% with at least a bachelor’s degree) White (9/10, 90%), Asian American (1/10, 10%), and none were Hispanic or Latino
Middelweerd et al (2020) [89] (Other related references: Klein et al [90] and Middelweerd et al [91])	The Netherlands	3-arm quasi-experimental; 12 weeks	Community (flyers, posters, social media, personal contacts, and snowball strategies)	104	Adults aged 18-30 years at the time of registration, in possession of a suitable smartphone running on Android or iOS, apparently healthy, Dutch-speaking, and signed the informed consent form	Healthy young adults Mean age 23.4 (SD 3.0) years Female (83/104, 79.8%) Students (72/104, 69.2%) Mean BMI 22.8 (SD 3.4) kg/m2 Previous experience with PA apps (33/104, 31.7%)
Korinek et al (2018) [92] (other related references: Freigoun et al [93] and Martin et al [22])	United States	Pre-post single-arm intervention; 14 weeks	Nationally via community advertising methods (eg, email to student listservs, word-of-mouth, and social media ads)	20	Generally healthy, insufficiently active, 40 to 65 years, BMI 25 to 45 kg/m^2^, owned and regularly used an Android phone capable of connecting to a Fitbit Zip via Bluetooth 4.0	Overweight and sedentary adults Age (47 years) Mean BMI 33.8 (SD 6.82) kg/m2 Female (18/20, 90%) Walked on average 4863 steps per day
Rabbi et al (2015) [94]	United States	Pilot RCT^b^; 3 weeks	Advertisement placed throughout the university campus	17 (intervention=9; control=8)	Owned an Android mobile phone, interested in fitness	Adult students and staff Mean age 28.3 (SD 6.96) years Student (13/17, 76%) Female (8/17, 47%) All participants (low-to-moderate PA)
Rabbi et al (2018) [95]	United States	Pilot Pre-post single-arm intervention; 5 weeks	Via the Wellness Center and retiree mailing lists from Cornell University	10	People with a history of chronic back pain (≥6 months in duration); willing to use MyBehaviorCBP; having some reasonable level of outdoor movement (eg, traveling to and from work); not being significantly housebound; with a basic level of mobile phone proficiency; aged between 18 years and 65 years; and fluent in English	Adults with chronic low back pain Mean age 41.1 (SD 11.3; range 31-60) years Female (7/10, 70%)
Zhou et al (2018) [96]	United States	RCT; 10 weeks	Email announcement; university campus	64 (intervention=34; control=30)	Staff member, intended to be physically active in the next 10 weeks; own an iPhone 5s or newer; willing to keep the phone in the pocket during the day; willing to install and use the study App; able to read and speak English	Adult staff employees Small fraction had the following conditions: high blood pressure (5/64, 8%), type 2 diabetes (5/64, 8%), hypercholesterolemia (7/64, 11%) Married or cohabitating (34/64, 56%) White or non-Hispanic (29/64, 45%) Full-time job (45/64, 70%) Mean age 41.1 (SD 11.3) years Female (53/64, 83%)

^a^PA: physical activity.

^b^RCT: randomized controlled trial.

### General Description of the Studies

A total of 5 studies were conducted in the United States [12,92,94-96], 1 in Italy [86], 1 in New Zealand [87,88], and 1 in the Netherlands [89-91]. Of these, 3 studies used pre-post intervention designs [87,92,95], 2 were RCTs [94,96], 1 was a 3-arm quasi-experimental study [89], 1 was a single-group microrandomized trial [12], and 1 was development study [86]. The duration of the studies ranged from 3 weeks to 6 months. A total of 5 studies recruited participants from community settings [12,86,87,89], 1 from the university campus and community [92], 2 from the university only [94,96], and 1 from a university wellness center and retiree mailing list [95]. Populations included insufficiently active and sedentary healthy adults, healthy and highly educated young adults, overweight and sedentary adults, adults with chronic low back pain, and students and staff from a university setting. Sample sizes ranged from 10 to 104 participants in the intervention evaluation studies and 60 in the development study. Participants were predominantly women in all studies except one [94]. The general characteristics of the included studies are summarized in Table 1.

### Theoretical Premise

SCT was the predominant psychological theory used [12,89,92,94,95]. A study incorporated self-efficacy theory [86], with a dynamic decision network—a sequence of simple Bayesian networks used to describe probabilistic computational models [97]. A study used an integrated behavior change model incorporating 33 behavior change techniques (eg, self-monitoring, goal setting, and review of goals) combined with the behavioral intervention technology model [87]. Two studies incorporated control systems engineering models integrated with SCT [12,92]. In a study, SCT, self-regulation theory, and health action process approaches were integrated with a computational agent model—an intelligent reasoning system [89]. Learning theory, the Fogg behavior model, and SCT were combined with the exploit-explore strategy in 2 studies [94,95]. Rather than using a theoretical framework, a study integrated a single behavior change technique (goal setting) with a behavioral analytic algorithm [96].

### Featured Description of Interventions

All studies promoted PA, whereas 4 studies also involved interventions for reducing SB [12,87,94,95]. Few studies explicitly stated the inclusion of behavior change techniques [87,89,92] as part of their intervention, 2 of which included a range of behavior change techniques [87,89]. Conroy et al [12] did not describe specific behavior change techniques but stated using intervention messages, which targeted key SCT constructs (eg, outcome expectancies, risk awareness and planning, efficacy-building affirmations, social support, and evoking anticipated reward or regret). The most common behavior change technique used across all studies was goal setting [86,87,89,92,96]. In terms of PA, 3 studies [87,92,96] included daily goal setting to achieve PA targets, whereas 1 study promoted weekly goal setting [89]. In a study, weekly step goals were initially established and then broken down into daily short-term goals [86]. Only 1 study set goals for SB [87]. To help participants set PA and SB goals, 5 studies used past activity performance [86,87,89,92,96], whereas 2 also took into account individuals’ perceptions of self-efficacy [86,92]. Instead of setting goals, 2 studies focused on habit formation by providing suggestions from an individual’s past frequent and infrequent activities after manual and automatic logging and clustering of past activities [94,95]. Habit formation was also accounted for in another study [87]. For SB, Direito et al [87] encouraged participants to replace periods of extended sedentary time with light-intensity walking and standing, whereas Conroy et al [12] included *sit less* and *move more* messages. Two other studies by Rabbi et al [94,95] targeted both SB and standing by promoting short walks. None of the studies measured standing as an outcome.

Monitoring and feedback on behavior was another widely used behavior change technique [86,87,89,92,94,96]. All 6 studies used visual and numerical feedback on behavior, whereas 2 used biofeedback to help monitor behavior [86,94]. Four studies included a reward in the form of social rewards [19,20,94] and material incentives [17]. In terms of the type of intervention, 2 studies used push notification messages [87,89], 3 used push notifications to present step goals or minutes of activity goals (eg, walking) [86,92,96], 2 had in-app suggestions selected from frequent and infrequent past activities [94,95], and 1 used text messages [12].

In total, 7 studies used mobile apps, 6 of which ran on Android [86,87,89,92,94,95] and 1 on iPhone operating systems (iOS) [96]; 1 study did not mention the operating system used [12]. Four studies including TODAY, MyBehavior, MyBehaviorCBP, and CalFit [87,94-96] used built-in smartphone sensors (ie, accelerometers) to measure activity, and 3 studies used wearable activity trackers (Fitbit One, Fitbit Zip, and ActivPAL3) [12,89,92]. A heart rate sensor was used to measure activity in the study by Baretta et al [86]. Furthermore, 3 studies used the phone GPS to identify geo-locations [89,94,95]. Some studies used built-in phone GPS and apps to capture and account for environmental contexts such as location (eg, workplace) [87,89,94,95], weather [89,92], and weekend or weekday [12,92]. The JustWalk intervention incorporated psychological states (eg, stress) and measures of busyness and sleep quality. Further details have been provided in Table 2.

**Table 2:** Features of smartphone-based physical activity intervention development or evaluation.

Author (year)	Intervention	Control	Theoretical premise	Primary outcome	Other outcomes	Technology feature	Results
Baretta et al (2019) [86]	Weekly tailored PA^a^ goals Starting goal (first week): 120 METb Long-term goal: 600 METs per week of PA Weekly goals broken down into daily goals Factors not considered in the intervention development but proposed for the next study: working hours, time of the day, day of the week, health and illness, weather, etc	N/A^c^	Self-efficacy theory and dynamic decision network	PA measured by HR^d^ sensor, self-efficacy beliefs	N/A	Android app: Muoviti (visualizing the heart-beat rate graph of the last training session, the curves of weight and waistline variations week by week, the burned calories graph, session by session, and the percentage of vigorous activity with respect to moderate activity) Other: HR wristbands (MioAlpha and PulseON)	N/A
Direito et al (2018 and 2019) [87,88]	Daily individualized and adaptive PA and SB^e^ goals: Daily activities (eg, transport to or from work, PA at work) Light-intensity activity to replace SB (eg, walking to a colleague’s desk rather than call or email, stand up while on the phone) Leisure-time moderate-to-vigorous PA (eg, cycling) Daily goals, visual and numerical feedback on past day and historical data, tips or suggestions, infographics, videos, and links, frequently asked questions, reminders, and push notifications Context: workplace (location)	N/A	Intervention mapping taxonomy to identify behavior change techniques (eg, self-monitoring, goal setting, or review of goals) from literature. Integrated behavior change model constructs and behavioral intervention technology; 33 behavior change techniques were included	Test the acceptability and feasibility of just-in-time adaptive intervention on PA and SB	Pilot-testing the TODAY^f^ app	Android apps: Art of Living app and TODAY app. Other: built-in phone sensors for SB and activity (ie, accelerometer)	TODAY app: low-effort and pleasant (54.3%), provides guidance on changing activity profile (52.6%), positively framed messages (64.4%), the app sustained interest over the 8 weeks (28.8%) Most favorable behavior change techniques for the users (goal setting, discrepancy between current behavior and goal, feedback on behavior, instruction on how to perform the behavior, and behavior substitution) Only significant improvement was occurred on light PA (see the results for statistics)
Conroy et al (2018) [12]	Five daily text messages (between 8 AM to 8 PM). Three message types (move more, sit less, general facts or trivia [unrelated to PA or SB]). Message receipt was confirmed with a reply. Factors: context (weekday and weekend)	N/A	Social cognitive theory and control systems engineering	Stepping time	N/A	No app or text message ActivPAL3 (activity tracker)	(Proof-of-concept study) 50% of the sample: more pronounced behavioral responses to text messages on weekends than weekdays; 50% had similar weekend or weekday responses; 50% of responders increased stepping time in response to “move more” messages, and 50% increased stepping time in response to “sit less” messages
Middelweerd et al (2020) [89], Klein et al (2017) [90], and Middelweerd et al (2018) [91]	Weekly moderate-to-vigorous PA goals: 30 minutes of moderate PA for at least 5 days a week or 20 minutes of vigorous PA for 3 days a week Contexts (location, weather, occupation) Connected friends (Facebook API^g^), if 2 participants of the intervention are connected Up to 3 messages a day	N/A	Social cognitive theory, self-regulation theory and health action process approach and computational agent model	To increase the total time spent in moderate-to-vigorous PA	N/A	Android app: Active2Gether Fitbit One (for self-monitoring only), ActiGraph wGT3XBT and GT3X+ (activity trackers)	No significant intervention effects were found for the Active2Gether-full and Active2Gether-ight conditions on levels of PA compared with the Fitbit condition: larger effect size for Active2Gether-ight (*β*=3.1, 95% CI −6.66 to 12.78, for minutes of moderate-to-vigorous PA; *β*=5.2, 95% CI −1334 to 1345, for steps). Smaller effect size for Active2Gether-full (*β*=1.2, 95% CI −8.7 to 11.1, for minutes of moderate-to-vigorous PA; *β*=−389, 95% CI −1750 to 972, for steps)
Korinek et al (2018) [92] and Freigoun et al (2017) [93]. More information is available in Martin et al (2018) [22]	Daily step goal: Pseudorandomly assigned daily step goal (doable [based on baseline median daily step] and ambitious [ie, up to 2.5×baseline median])+ rewards (points>Amazon Gift Cards) Six 16-day cyclesh (cycle 0 [baseline], cycles 1 to 5 [step goals assigned]) Step goals prompted every morning+there were daily, weekly and monthly surveys Morning and evening EMAi assessed constructs including (eg, confidence in achieving the goal, predicted busyness for that day, previous night’s sleep quality) Factors considered: perceived stress, perceived busyness, weather information, sleep quality	N/A	Social cognitive theory (particularly self-efficacy construct), goal setting and control systems engineering (system identification)	Feasibility, daily steps	N/A	Android app: JustWalk Fitbit Zip (activity tracker) Other: web-based mobile questionnaire	Linear mixed effect model: each individual walked below 5000 steps at baseline with significant variation; mean intercept value 4863.3 steps (SD 1838.42), *t*_98_=10.49; *P*<.001. Daily steps increased by 2650 steps per day on average from day 0 to day 16 (cycle 0 to cycle 1); *t*_98_=6.54, *P*<.001. Quadratic mixed effect model: each individual walked roughly 5000 steps at baseline with significant variations; mean intercept value 5301.5 steps (SD 1862.04); *t*_98_=11.29, *P*<.001. Daily steps increased by 1500 steps per day on average from cycle 0 to cycle 1 (1505 steps; *t*=5.52, *P*<.001); however, daily steps decreased by 247.3 steps per day on average from day 0 to day 16 (cycle 0 to cycle 1); *t*_98_=-5.01, *P*<.001 High adherence was observed (only 10 days of having missing step data; only 40 days of nonwear; <500 step counts). Common problem: sync lag with Fitbit
Rabbi et al (2015) [94]	Daily personalized context-sensitive suggestions (PA and stationery). Manual and automatic logging to track activity and user location. Start of each day: 10 in-app activity suggestions (90% users’ most frequent activities [exploit]; 10% from users’ infrequent activities [explore]). MyBehavior app included both PA and dietary interventions	Nonpersonalized generic recommendations	Learning theory, Fogg behavior model, social cognitive theory, and exploit-explore strategy^j^	Adherence, acceptability, behavior change	N/A	Android app: MyBehavior; other: phone accelerometer and GPS	Intervention participants more intended to follow personalized suggestions than control (effect size=0.99, 95% CI 0 to 1.001; *P*<.001). Most intervention participants (78%) had a positive trend in walking behavior (also increased daily walking by 10 minutes during the intervention), whereas most control participants (75%) showed a negative trend. The users found MyBehavior app suggestion very actionable and wanted to follow them
Rabbi et al (2018) [95]	Context-sensitive suggestions (PA and stationery). Manual and automatic logging to track activity and user location. In-app suggestions (80% users’ most frequent activities [exploit]; 20% from users’ infrequent activities [explore]); total time for each selected activity must not exceed 60 minutes. End of day reward score	Static suggestions	Learning theory, Fogg behavior model, social cognitive theory (self-efficacy) and exploit-explore strategy^j^	Use, acceptability, early efficacy	Qualitative feedback	Android app: MyBehaviorCBP; other: phone accelerometer and GPS	Intervention condition increased daily walking by 4.9 minutes (*β*=4.9; *P*=.02) significantly. Exercise time was increased nonsignificantly by 9.5 minutes (*β*=9.5; *P*=.31). MyBehaviorCBP was opened 3.2 times a day (on average). MyBehaviorCBP suggestions were perceived as low-burden (*β*=.42; *P*<.001). Back pain was reduced in the intervention condition, but not significantly (*β*=−.19; *P*=.24). Participants suggested consideration of weather, weekend or weekday, and level of pain for future interventions
Zhou et al (2018) [96]	Daily step goals (real-time, automated adaptive). Push notifications via app. Daily notifications at 8 AM. If the goal was accomplished before 8 PM, a congratulation notification was sent.	Steady step goals (10,000 per day)	Goal setting and behavioral analytics algorithm^k^	Change in daily step	Step goal attainment, weight, height, barriers to being active quiz, IPAQ^l^-short form	iOS app: CalFit; other: built-in health chip in the iPhone	Mean daily step count was decreased by 390 steps (SD 490) per day in the intervention versus 1350 steps (SD 420) per day in the control from baseline to 10 weeks (net difference: 960 steps, *P*=.03)

^a^PA: physical activity.

^b^MET: metabolic equivalents.

^c^N/A: not applicable.

^d^HR: heart rate.

^e^SB: sedentary behavior.

^f^TODAY: Tailored Daily Activity.

^g^API: application programming interface.

^h^Step goals did not increase between cycles.

^i^EMA: ecological momentary assessment.

^j^Grounded in artificial intelligence and a subcategory of a broader decision-making framework called multiarmed bandit, which stems from probability theory.

^k^Behavioral analytics algorithm uses machine learning to build a predictive model–based on historical and goal steps for a particular person and then uses this estimation to generate challenging yet realistic and adaptive step goals based on a predictive model that would maximize the physical activity in the future.

^l^IPAQ: International Physical Activity Questionnaire.

### Effectiveness of Interventions

#### Narrative Synthesis of Individual Studies

A total of 6 studies reported on the effectiveness of the intervention [87,89,92,94-96]; the details are presented in Table 1. The intervention by Direito et al [87] increased the time spent in light and moderate-to-vigorous intensity PA and total PA time; however, only light-intensity PA achieved statistical significance from pre- to postintervention assessments (adjusted mean difference 2.2 minutes, SE of difference 1.0; 95% CI 0.78-3.56; *P*=.002). A small, but statistically nonsignificant, decrease in SB was observed (adjusted mean difference −9.5 minutes, SE of difference 7.5; 95% CI 19.98-1.05; *P*=.08). The Active2Gether intervention involved 3 arms of Active2Gether-full (tailored coaching messages, self-monitoring, and social comparison), Active2Gether-light (self-monitoring and social comparison), and Fitbit app control condition (self-monitoring). The Active2Gether did not show an effect on PA levels (average daily minutes of MVPA and step counts) compared with the Fitbit app (*β*=1.2, 95% CI −8.7 to 11.1, *P*=.81, for minutes of MVPA; *β*=−389, 95% CI −1750 to 972, *P*=.57, for steps and *β*=3.1, 95% CI −6.66 to 12.78, *P*=.53, for minutes of MVPA; *β*=5.2, 95% CI −1334 to 1345, *P*=.99, for steps, for the full and light app, respectively). The JustWalk intervention increased the average daily steps by 2650 steps in 16 days (*t_98_*=6.54; *P*<.001). This effect decreased from day 16 to day 96 (average daily change −109.1 steps; t_98_=−1.42; *P*=.15), suggesting acceptable maintenance. Users of the MyBehavior app walked an average of 10 minutes per day more from the first to the third week. There was no change in the control group (between-group differences were statistically significant (*t_15_*=2.1; *P*=.06; 95% CI −0.23 to 19.05; *d*=0.9). In the second study by Rabbi et al [95], MyBehaviorCBP was associated with an increased daily walking time of 4.9 minutes (*β*=4.9; *P*=.02; 95% CI 0.8-0.89; *d*=0.31) among adults with chronic back pain. Nonwalking exercise time also increased by 9.5 minutes, but it was not statistically significant (*β*=9.5; *P*=.31; 95% CI −6.3 to 21.8; *d*=0.03). The Cal Fitness trial showed that the mean daily step count decreased in the 10-week intervention for both the intervention (mean −390, SD 490) and control group (mean −1350, SD 420; net mean difference 960; 95% CI 90-1830; *P*=.03). The Conroy et al [12] study was conducted to determine proof-of-concept and did not report effectiveness data (for descriptive results, see Table 2).

#### Effect Direction Plot

**Figure 2:**
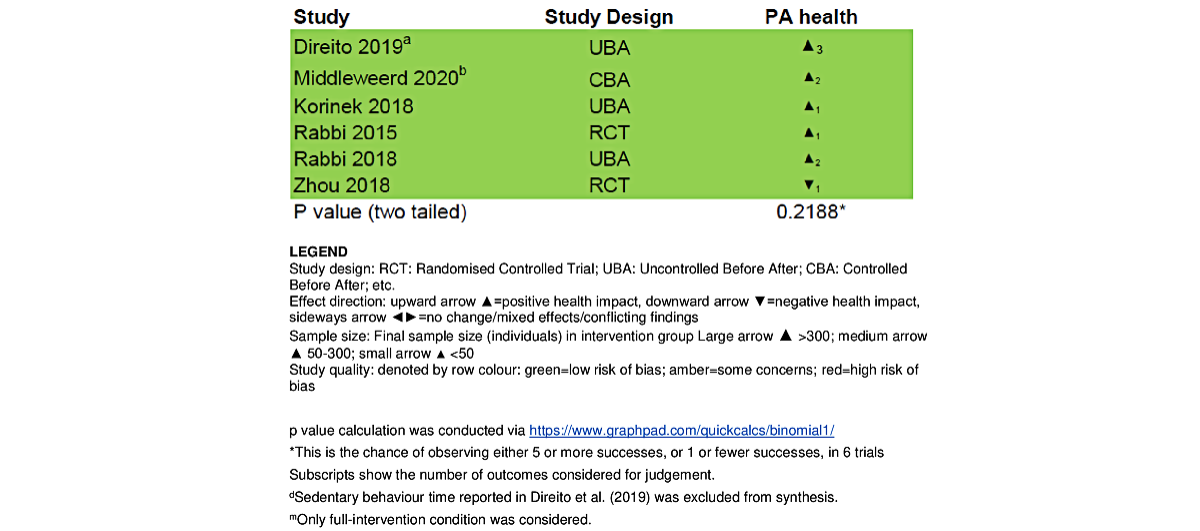
Effect direction plot summarizing the direction of impact from smartphone-based physical activity interventions.

This study included 6 interventional studies. Figure 2 shows the effect direction plot for the PA health outcome domain; 5 of 6 interventions reported a positive effect direction, with 1 study showing a negative effect on PA health. The *P* value for the sign test for PA health was *P*=.21.

### Risk of Bias Assessment of the Included Interventions

Judgments on the risk of bias for the 2 RCTs and 4 quasi-experimental studies are presented in Multimedia Appendix 3 [12,87,89,92,94-96]. Overall, the included studies were of relatively high quality. The 2 RCTs [94,96] were judged to be low risk in all domains except one (ie, blinding of participants and personnel). All included quasi-experimental studies lacked a control group because of a pre-post [87,92,95] or single-group intervention [12] design. These interventions did not introduce additional risks to the remaining eight domains.

## Discussion

### Principal Findings

This review aims to quantify the number of studies that have integrated traditional psychological theories with dynamic computational models in the development or evaluation of smartphone interventions to reduce SB and promote PA. Although we showed that a few studies—mainly pilot, feasibility, and proof-of-concept—have taken an integrated dynamic approach, there was no consensus on what dynamic model–based approach should be used and how. Overall, it was difficult to draw a conclusion on the effectiveness of the included smartphone interventions; however, preliminary findings on PA are promising, less so for SB. Moreover, an effect direction plot was used to illustrate the direction of the intervention effect on PA outcomes, regardless of their statistical significance.

This review was driven in part by a paper by Riley et al [4] who argued that to truly capture the benefits of smartphones to deliver real-time and adaptive interventions, they need to adopt principles from other disciplines, such as control systems engineering, and integrate them with traditional health behavior theories. In total, we found only 8 studies that had adopted this notion, most of which used SCT for integration, with considerable complexity in the approaches used, ranging from a basic use of behavioral analytic algorithms to a more sophisticated approach using control systems.

Advancements in smartphone technology have enabled the collection of intensive contextual and longitudinal (time-variant) data, which facilitate the delivery of automated, real-time, and adaptive behavior change interventions such as just-in-time adaptive interventions. These features permit the testing of specific intervention components (eg, behavioral messaging comprising behavior change techniques). Control systems appear to offer an excellent fit for the development of adaptive smartphone interventions. It explores ways to influence a dynamic system (eg, time-varying adaptive PA intervention) and how to regulate it [11,12]. In other words, control systems engineering provides a dynamic approach to designing tailored interventions that adapt over time and are based on real-time data (ie, intensive longitudinal data) [98]. Despite the variability in the application of dynamic models outlined in this review, existing evidence suggests that their integration with traditional behavior change and psychological theories offer exciting opportunities to better understand human behavior (eg, SB and PA), identify patterns of behavior, and optimize individually adapted behavior change interventions.

Few of the included studies evaluated the effectiveness of the interventions, and small effects were observed on PA and SB. Possible reasons for the small effect sizes may have included inappropriate design (nonrandom allocation) [89], lack of exposure to automated intervention because of technical problems [89], use of nonpersonalized behavioral interventions [94], lack of participant engagement with the intervention [87], and insufficient inclusion of behavior change techniques [96]. Moreover, a binary sign test conducted in this review attempts to provide additional information and contribute to transparency in interpreting the effect direction. However, this should be interpreted carefully, as the small number of studies may have underpowered the test.

Most of the studies included in the review focused on PA, whereas only a few targeted SB; none assessed standing as a distinct outcome. Moreover, most smartphone-based SB and PA interventions used built-in smartphone accelerometers and sensors as a tool to capture individual behaviors to inform behavioral interventions (ie, step counts were used to help participants set goals and monitor progress or provide activity suggestions) [87,94-96].

The benefits of smartphone interventions include the ability to collect and measure contextual factors (eg, location, weather, and emotional or psychological states), which could be used to personalize behavior interventions [99]. Existing research evidence has shown that contextually aware reminders increase the effectiveness of mHealth PA interventions [21,100]. Furthermore, leveraging contextual information in PA interventions enables the triggering of more frequent reminders without annoying the individual receiving the reminder, and these types of interventions are considered more acceptable [100]. Despite these proposed benefits, most of the included studies lacked an assessment of contextual factors. A likely reason for the lack of contextual factors in the reviewed studies is the technical challenges, such as system requirements. For example, high battery consumption and low localization speed by a built-in smartphone GPS compromise mobile app performance [101]. Another important reason might be the privacy implications for smartphone users [102]. Privacy breaches are most probable when context-sensitive information such as location is monitored [103]. Moreover, people generally refuse to be monitored for where they go or what they do [104]. A limitation of using native smartphone sensors is that they do not provide research-grade precision for measuring PA and SB. Commonly used accelerometers (eg, built-in smartphone accelerometers and Actigraph GT3X) measure SB by focusing on periods where the device records activity counts below a certain cutoff point, such as less than 100 counts per minute [105]. This leads to the miscategorization of SB [106]. Although postural devices (inclinometers) such as activPAL have excellent accuracy in measuring SB [107], they require proprietary software (activPAL Professional Research Edition, PAL Technologies) to process and collect the data and thus have low utility for real-time interventions. Finally, as highlighted above, none of the included studies assessed standing as an outcome, despite 3 studies promoting standing in their intervention messages [87,94,95]. This might be explained by the inability to measure standing in real time for a dynamic intervention purpose and limited evidence advocating standing as a distinct activity that brings health benefits. However, short-term and small-scale studies that support standing are emerging. In a lab-based study, breaking up every 30 minutes of sitting by 5 minutes of standing was shown to reduce postprandial blood glucose (34% reduction) compared with prolonged sitting in postmenopausal women [108]. Moreover, an office-based study has shown that an afternoon of standing reduced postprandial glucose (43% reduction) compared with sitting while performing computer work [109].

The included interventions comprised pre-post, RCT, and 3-arm quasi-experimental designs. These commonly used experimental designs are unable to assess rich context and time-intensive data. For example, RCTs do not provide information on the particular time when the intervention had an effect and the moderators that affected the behavior change [110]. In fact, RCTs typically consider the overall impact of an intervention package on behavior or health outcomes, not specific components of that intervention. Other study designs, such as factorial designs, are capable of investigating the effects of each intervention component and the interactions between components and the dosing of the intervention. However, they are not sufficient to delineate when the intervention was most effective and what moderators influenced the intervention [110]. A microrandomized trial may address these design limitations. The microrandomized trial is a novel experimental design to determine the optimal delivery of just-in-time adaptive interventions [110]. A key advantage is that microrandomized trials not only assess the effect of specific intervention components but also changes in effects over time and moderators, including contextual and psychological factors [110]. Microrandomization can help elucidate potential causal relationships between each randomized intervention feature and proximal effects (what happens in a limited time window, for example within 1 hour, following a randomized intervention) and allow assessment of time-varying contextual and psychological factors moderating those proximal effects [110].

Most of the included studies lacked comprehensive incorporation and testing of behavior change techniques, although they were theory-based. The precise specification of behavior change techniques—which are active ingredients of behavior change interventions and specification of intervention features of PA (eg, mode of delivery and frequency)—help provide accumulative evidence for effective and replicable interventions [111]. Smartphone-based interventions undertaking dynamic approaches with a proper experimental design (ie, microrandomized), while testing various behavior change techniques, are expected to provide more robust evidence than traditional theory approaches.

### Limitations and Strengths

A limitation of this review is the heterogeneity in the reported effectiveness data that prevented a pooled meta-analysis. Other limitations include the small sample size and short duration of the included interventions and nonrandomized study designs. Moreover, women exceeded men in most studies, and all studies involved adult populations, which might limit the generalizability of the findings. A key strength of this review is that it focuses on the integration of dynamic models in smartphone-based PA and SB studies, as such dynamic models fit best with mobile technologies. Another strength is the use of the effect direction plot to present the direction of the effectiveness results. This methodology is superior to narrative synthesis, as it helps with the overall interpretation of the findings. Future studies, in the context of SB and PA behaviors, are suggested to incorporate and assess the effect of relevant environmental and internal contextual moderators, use computational models, and investigate SB, in particular, as there is a significant evidence gap.

### Conclusions

In conclusion, despite the recommendation for integrating dynamic models such as control systems to better harness the potential of mobile technologies, this review showed that few studies have actually adopted this approach to promote PA and reduce SB. To some extent, this research gap may be because of the complex and multifaceted nature of dynamic models, such as control systems, in integrating adaptive contexts and real-time measurement of outcomes.

## Appendix

Multimedia Appendix 1 Search strategy.

Multimedia Appendix 2 Reason for exclusion.

Multimedia Appendix 3 Quality assessment.

## Abbreviations

mHealth: mobile health
MVPA: moderate-to-vigorous physical activity
PA: physical activity
PRISMA: Preferred Reporting Items for Systematic Reviews and Meta-Analyses
PROSPERO: International Prospective Register of Systematic Reviews
RCT: randomized controlled trial
SB: sedentary behavior
SCT: social cognitive theory
