# Supplementary material for: Smartphone-Based Interventions to Reduce Sedentary Behavior and Promote Physical Activity Using Integrated Dynamic Models: Systematic Review
Source: J Med Internet Res. 2021 Sep 13;23(9):e26315. doi: 10.2196/26315 (PMC8477296; doi:10.2196/26315)
Supplement: Multimedia Appendix 1 [file jmir_v23i9e26315_app1.docx]

**Multimedia Appendix 1. search strategy.**

Keywords (including MeSH terms) and phrases were incorporated into three components, where “OR” and “AND” Boolean operators used for within and between component/s searching, as follows:

Component 1 ([**OR**] “mobile health”, “m-health”, mhealth, phone, smartphone, cellphone, telehealth, telemedicine, app, apps, application*)

**AND**

Component 2 ([**OR**] “control systems engineering”, “control model”, “control theory”, “system identification”, “hybrid model predictive control”, “adaptive model”, “adaptive models”, “adaptive system”, “adaptive systems”, “personalized model”, personalized models”, “decision trees”, “system performance”, “dynamic task-redistribution”, “context-aware adaptation”, “contextually aware”, “contextual aware”, “adaptive middleware”, “performance optimization”, “performance optimization”, “mobile computing”, “context awareness”, “decision support system”, “analytical models”, “context modeling”, “context modelling”, “personalized medicine”, “precision medicine”, “model predictive control”, “recommender systems”, “recommender system”, “agent-based modeling”, “agent-based modelling”, “Bayesian network”, algorithm*, “just-in-time”, “machine learning”, “micro-randomized”, “micro-randomised”, “microrandomized”, “microrandomised”, “micro-randomization”, “micro-randomisation”, “sequential randomization”, “sequential randomisation”, “automated personalized feedback”, “hybrid design”, “real-time”, “rich context”, “agent-based”, “just-in-time”, “ecological momentary assessment”, “ecological momentary intervention”, “ecological framework”, “integrated behaviour change model”, “integrated behaviour change model”, “integrated model”, “cyber physical system”, “adaptive”, computational, “dynamic feedback system”, “dynamical system”, “dynamic system”, “dynamical model”, “dynamic model”, “production-inventory”, “adaptive controller”, “artificial intelligence”, “deep learning”, “neural networks”)

**AND**

Component 3 ([**OR**] “physical activity”, exercis*, walk*, jog*, sedentary, sitting)

The wild-card term "*" was used where necessary to potentiate sensitivity.

Snowball searching were performed among included studies to find further relevant research.
