# Supplementary material for: Smartphone-Based Interventions to Reduce Sedentary Behavior and Promote Physical Activity Using Integrated Dynamic Models: Systematic Review
Source: J Med Internet Res. 2021 Sep 13;23(9):e26315. doi: 10.2196/26315 (PMC8477296; doi:10.2196/26315)
Supplement: Multimedia Appendix 2 [file jmir_v23i9e26315_app2.docx]

**Multimedia Appendix 2. Reason for exclusion:**

| **Item** | **Author/year** | **Reason** |
| --- | --- | --- |
| 1 | Adams et al., 2013 | Not smartphone-based |
| 2 | Bickmore et al., 2011 | Not smartphone-based |
| 3 | Hurling et al., 2007 | Not smartphone-based |
| 4 | Fukuoka et al., 2011 | Not intervention development or evaluation (qualitative study) |
| 5 | Hughes et al., 2010 | Not intervention development or evaluation (introducing technology) |
| 6 | Liao et al., 2018 | Not intervention development or evaluation (study examples) |
| 7 | Martin et al., 2018 | Not intervention development or evaluation (model simulation) |
| 8 | Pirolli et al., 2018 | Not intervention development or evaluation |
| 9 | Yang et al., 2018 | Not intervention development or evaluation |
| 10 | Stroulia et al., 2013 | No intervention development or evaluation |
| 11 | Naved & Uddin, 2018 | Not intervention development or evaluation |
| 12 | Zheng et al., 2010 | Not intervention development or evaluation |
| 13 | Pirolli et al., 2016 | Not intervention development or evaluation (model representation) |
| 14 | Zhao et al., 2013 | Not intervention development or evaluation |
| 15 | Huang et al., 2018 | Not intervention development or evaluation |
| 16 | Muller et al., 2017 | No intervention development or evaluation |
| 17 | Mohadis et al., 2016 | Not dynamic model |
| 18 | Boulton et al., 2019 | Not dynamic model |
| 19 | Martin et al., 2015 | Not dynamic model |
| 20 | Buman et al., 2016 | Not dynamic model |
| 21 | Lim et al., 2016 | Not dynamic model |
| 22 | Rawstorn et al., 2016 | Not dynamic model |
| 23 | Vankipuram et al., 2012 | Not dynamic model |
| 24 | Martin et al., 2016 | Not dynamic model |
| 25 | Ryan et al., 2018 | Not dynamic model |
| 26 | Clark et al., 2018 | Not dynamic model |
| 27 | Klasnja et al., 2018 | Not dynamic model |
| 28 | Bond et al., | Not dynamic model |
| 29 | Marquet et al., 2018 | Assessment, not intervention |
| 30 | Srinivas et al., 2019 | Assessment, not intervention |
| 31 | Cruciani et al., 2017 | Assessment, not intervention |
| 32 | Dunton et al., 2016 | Assessment, not intervention |
| 33 | Dunton et al., 2014 | Assessment, not intervention |
| 34 | Dunton et al., 2012 | Assessment, not intervention |
| 35 | Rofey et al., 2010 | Assessment, not intervention |
| 36 | Smith et al., 2017 | Assessment, not intervention |
| 37 | Jones et al., 2016 | Assessment, not intervention |
| 38 | Li et al., 2019 | Assessment, not intervention |
| 39 | Fahim et al., 2018 | Assessment, not intervention |
| 40 | Brannon et al., 2016 | Assessment, not intervention |
| 41 | Valentiner et al., 2019 | Assessment via mobile App; intervention via phone call |
| 42 | Alian et al., 2018 | Case study |
| 43 | Hales et al., 2016 | Not adaptive design |
| 44 | Salvi et al., 2018 | Not adaptive design |
| 45 | Tabak et al., 2014 | Not adaptive design |
| 46 | Maddison et al., 2019 | Not adaptive design |
| 47 | Kramer et al., 2019 | No intervention delivery via smartphone (SMS) |
| 48 | Militello et al., 2016 | No intervention delivery via smartphone (SMS or face-to-face) |
| 59 | Brakenridge et al., 2018 | No intervention delivery via smartphone |
| 50 | Yom-Tov et al., 2017 | Intervention delivered via SMS |
| 51 | Lin et al., 2015 | No intervention delivery via smartphone (passive use of App) |
| 52 | Alexander et al., 2017 | No intervention delivery via smartphone (SMS messaging) |
| 53 | Al-Ozairi et al., 2018 | Protocol |
| 54 | Baert et al., 2018 | Protocol |
| 55 | Bardus et al., 2018 | Protocol |
| 56 | Evans et al., 2015 | Protocol |
| 57 | Goldstein et al., 2018 | Protocol |
| 58 | Hassoon et al., 2018 | Protocol |
| 59 | Hurley et al., 2015 | Protocol |
| 60 | Orme et al., 2016 | Protocol |
| 61 | Pellegrini et al., 2014 | Protocol |
| 62 | Philips et al., 2018 | Protocol |
| 63 | Symons et al., 2018 | Protocol |
| 64 | Vidmar et al., 2019 | Protocol |
| 65 | Block et al., 2015 | No intervention delivery via smartphone (web-based, automated phone call, etc.) |
