# Supplementary material for: Smartphone-Based Interventions to Reduce Sedentary Behavior and Promote Physical Activity Using Integrated Dynamic Models: Systematic Review
Source: J Med Internet Res. 2021 Sep 13;23(9):e26315. doi: 10.2196/26315 (PMC8477296; doi:10.2196/26315)
Supplement: Multimedia Appendix 3 [file jmir_v23i9e26315_app3.docx]

**Multimedia Appendix 3. Quality assessment:**

Randomized studies

| Author/Year | random sequence generation | allocation concealment | blinding of participants and personnel | blinding of outcome assessment | incomplete outcome data | selective reporting | other sources of bias |
| --- | --- | --- | --- | --- | --- | --- | --- |
| Rabbi 2015 | low risk | low risk | high risk | low risk | low risk | low risk | low risk |
| Zhou 2018 | low risk | low risk | high risk | low risk | low risk | low risk | low risk |

Non-Randomized studies

| Author/Year | cause-effect | comparison similarity | similar treatment | control group | multiple measurements | complete follow-up | same measurements for any group | reliable outcome measurement | appropriate statistical analysis |
| --- | --- | --- | --- | --- | --- | --- | --- | --- | --- |
| Direito 2019 | yes | yes | yes | no | yes | yes | yes | yes | yes |
| Korinek 2018 | yes | yes | yes | no | yes | yes | yes | yes | yes |
| Conroy  2019 | yes | not applicable | not applicable | no | yes | yes | not applicable | yes | yes |
| Rabbi  2018 | yes | yes | yes | no | yes | yes | yes | yes | Yes |
| Middelweerd  2020 | yes | no | yes | yes | yes | yes | yes | yes | yes |
